# A new rare species of the *Rhadinaeadecorata* group from the Sierra Madre del Sur of Guerrero, Mexico (Squamata, Colubridae)

**DOI:** 10.3897/zookeys.780.25593

**Published:** 2018-08-08

**Authors:** Uri Omar García-Vázquez, Carlos J. Pavón-Vázquez, Jean Cristian Blancas-Hernández, Epifanio Blancas-Calva, Eric Centenero-Alcalá

**Affiliations:** 1 Laboratorio de Sistemática Molecular, Unidad Multidisciplinaria de Investigación Experimental, Facultad de Estudios Superiores Zaragoza, Universidad Nacional Autónoma de México. Batalla 5 de Mayo s/n, Col. Ejército de Oriente, Ciudad de México 09230, México; 2 Museo de Zoología and Departamento de Biología Evolutiva, Facultad de Ciencias, Universidad Nacional Autónoma de México. Apartado Postal 70-153, Ciudad de México 04510, México; 3 Colegio de Biólogos de Guerrero A.C. Calle Laureles No. 69, Colonia Los Sauces. Chilpancingo, Guerrero 39060, México; 4 Instituto de Investigación Científica Área de Ciencias Naturales, Universidad Autónoma de Guerrero. Av. Lázaro Cárdenas s/n, interior del Jardín Botánico, Ciudad Universitaria, Chilpancingo, Guerrero 39087, México; 5 Current address: Division of Ecology and Evolution, Research School of Biology, Australian National University. 46 Sullivans Creek Road, Canberra, Australian Capital Territory 2601, Australia

**Keywords:** Description, reptile, snake, systematics, taxonomy

## Abstract

A new species of the *Rhadinaeadecorata* group is described based on two specimens from the Sierra Madre del Sur, Guerrero, Mexico. The new species differs from all other members of the genus *Rhadinaea* by having: eight supralabials; 149–151 (male) ventrals; 63–77 (male) subcaudals; two large pale nuchal blotches, forming an incomplete collar that occupies two scales laterally and is bissected along the dorsal midline; a postocular pale marking consisting of a well-defined, narrow line beginning behind the upper posterior margin of the eye and extending posteriorly nearly horizontally until connecting with the nuchal blotches; and the dark ground color of the flanks extending to the lateral portion of the ventrals. The large nuchal blotches distinguish the new species from the other members of the *R.decorata* group, except for *R.cuneata* and some individuals of *R.hesperia* (pale nuchal marking one-scale wide in *R.marcellae*, absent in the other species). The condition of the postocular pale marking distinguishes it from *R.cuneata* and *R.hesperia* (postocular pale marking wedge-shaped in *R.cuneata*, not connected with the pale post-cephalic markings in *R.hesperia*). Furthermore, the number of subcaudals and the coloration of the lateral portion of the ventrals distinguish it from *R.omiltemana* and *R.taeniata*, the remaining congeners found in Guerrero (85–90 in males of *R.omiltemana* and 91–121 in *R.taeniata*; dark color of the flanks not reaching ventrals in the former species, occasionally and faintly in *R.taeniata*). Additionally, a new combination for *R.stadelmani* is proposed. The new species is the first described in the genus *Rhadinaea* in more than 40 years.

## Introduction

Snakes of the genus *Rhadinaea* Cope, 1863 (Colubridae: Dipsadinae) are distributed throughout Mesoamerica, ranging from the Sierra Madre Occidental of southern Sinaloa and Sierra Madre Oriental of northern Nuevo León in Mexico to northwestern Ecuador in South America, with an isolated species, *R.flavilata* (Cope, 1871), in the southeastern USA (Myers 1974). According to Myers (2011), the genus contains 20 species arranged in six species groups, mainly on the basis of their external morphology. These groups are (number of species in each group in parentheses) the *R.calligaster* (1), *R.decorata* (11), *R.flavilata* (2), *R.taeniata* (3), and *R.vermiculaticeps* (3) groups. Five species described after the publication of Myers’s (1974) revision and originally placed in *Rhadinaea* have been transferred to *Rhadinella* (Myers 2011). Thus, no new species of *Rhadinaea* (sensu Myers 2011) has been described since Myers’ systematic monograph of the group in 1974.

The *Rhadinaeadecorata* group is characterized by the following combination of traits (Myers 1974): the hemipenis is single, without special features; a subpreocular is usually present; supracloacal ridges are usually present in males. The body is either striped or lined, with at least a hint of a narrow, linear dark marking involving dorsal scale rows 4 or 5, occasionally bordered above by a pale streak or series of small pale spots. A pale postocular marking extends from, or lays a short distance behind, the upper rear edge of the eye. The line may extend horizontally toward the neck or obliquely toward the corner of the mouth. Dorsal scales are arranged in 17-17-17 rows. Ventrals are 110–175 in males and 114–186 in females. Subcaudals are 56–137 in males and 60–120 in females. The tail comprises between 25% and 48% of the total length.

The *Rhadinaeadecorata* group is the most diverse assemblage within the genus. Members of the group collectively range from the Mexican states of Tamaulipas and Sinaloa south and east to northwestern Ecuador, where they are mainly found in high mountains. All the species occur in Mexico and only *R.decorata* (Günther, 1858) is not endemic to the country (Myers 1974): *R.bogertorum* Myers, 1974 is known from northern Oaxaca (Myers 1974); *R.myersi* Rossman, 1965 from eastern Guerrero and southern Oaxaca (García-Vázquez et al. 2009); *R.macdougalli* Smith & Langebartel, 1949 from northern Oaxaca with an isolated population from Los Tuxtlas, Veracruz (Myers 1974, Pérez-Higareda et al. 2002); *R.cuneata* Myers, 1974 and *R.forbesi* Smith, 1942 from central Veracruz (Myers 1974); *R.marcellae* Taylor, 1949 from southern San Luis Potosí, Hidalgo, and northern Puebla (Nieto-Montes de Oca and Mendelson III 1997); *R.quinquelineata* Cope, 1886 from Hidalgo and northern Puebla (Myers 1974); *R.gaigeae* Bailey, 1937 from southern Tamaulipas, San Luis Potosí, Querétaro, and northern Hidalgo (Myers 1974); *R.montana* Smith, 1944 from central Nuevo León and western Tamaulipas (Myers 1974, García-Vázquez 2012); *R.hesperia* Bailey, 1940 from the Sierra Madre del Sur of Guerrero and Oaxaca, the Pacific coast from southeastern Sinaloa to Oaxaca, and the Balsas Basin of Morelos and Puebla; and *R.decorata* from the Atlantic coast of southeastern San Luis Potosí, Mexico, southwards into northwestern Ecuador (Myers 1974).

During fieldwork conducted in the Sierra Madre del Sur, Guerrero, between 2006 and 2008 we collected an unusual individual of *Rhadinaea* in the vicinity of El Molote. In eleven trips to the locality we were unable to locate another specimen. Eight years after concluding our fieldwork another specimen with similar characteristics was collected ca. 3 km NW (in straight line) of where the first specimen was found. The snakes show a unique combination of characters and are apparently allopatric with respect to closely related species of *Rhadinaea* (see comparison section). These two individuals possess a unique combination of characteristics leading us to conclude they represent a new species that we describe below.

## Materials and methods

Acronyms for herpetological collections follow Sabaj (2016), except for MZFC-HE for the Museo de Zoología of the Facultad de Ciencias, Universidad Nacional Autónoma de México. The specimens of the new species were fixed in 10% buffered formalin, subsequently transferred to 70% ethanol for permanent storage, and deposited in the herpetological collection of the MZFC-HE. We compared the new species with all the other species of *Rhadinaea*, based on the examination of 68 specimens belonging to 11 species, and also benefitted from data contained in the relevant literature (i.e. Myers 1974, Holm and Cruz 1994, Mendelson III and Kizirian 1995, Nieto-Montes de Oca and Mendelson III 1997, McCranie 2006, Myers 2011). We provide a list of the specimens examined in Supplementary material 1.

We follow Myers (1974) for scale nomenclature. We performed scale counts under a dissecting microscope. We counted the ventrals as suggested by Myers (1974). We scored bilateral characters on both sides. When the condition of a given character was not identical on both sides, we give the conditions on the left and right sides, in that order, separated by a slash (/). We recorded measurements with a ruler (nearest 1 mm), digital callipers (nearest 0.1 mm), or an ocular micrometer to the nearest 0.1 mm. We measured head length from the tip of the snout to the angle of the jaw. We measured all scale dimensions at their maximum. We examined the hemipenial morphology in the new species after removal of the right hemipenis from the preserved holotype. The hemipenes were partially everted in the preserved specimens and therefore we could not record characters that are only visible in the retracted organs. We followed Pesantes (1994), Myers and Cadle (2003), Zaher and Prudente (2003), and Angarita-Sierra (2014) for hemipenial preparation. We follow Zaher (1999) and Myers and McDowell (2014) for hemipenial morphological terminology.

## Results

Our review of the literature revealed the need to update the binomial name of *Rhadinaeastadelmani* Stuart & Bailey, 1941. The species was placed in the synonymy of *Rhadinaeahempsteadae* Stuart & Bailey, 1941 (= *Rhadinellahempsteadae*) by Myers (1974), but Mendelson III and Kizirian (1995) resurrected it based on additional material. Later, Myers (2011) resurrected the genus *Rhadinella* Smith, 1941 to accommodate the former members of the *Rhadinaeagodmani* group, including *Rhadinellahempsteadae*. However, he did not comment on the generic placement of *Rhadinaeastadelmani*. Given the apparent close relationship of *Rhadinaeastadelmani* and *Rhadinellahempsteadae* and the morphological agreement of *Rhadinaeastadelmani* with the diagnosis of *Rhadinella* (Stuart and Bailey 1941, Myers 1974, 2011, Mendelson III and Kizirian 1995), we propose a new combination for *Rhadinaeastadelmani* as follows: *Rhadinellastadelmani* (Stuart & Bailey, 1941), comb. n.

Morphological examination of the specimens from El Molote supported their inclusion in the genus *Rhadinaea* (sensu Myers 2011), based on the following combination of traits (Myers 1974): hemipenis symmetrical, distally calyculate, unicapitate, spinose; sulcus spermaticus bifurcate; pupil round; full complement of colubrid head plates; subpreocular present; dorsal scales smooth, arranged in 17 rows with no posterior reduction; head with distinctive markings; body brown with longitudinal dark lines. Additionally, the specimens agree with Myers’s (1974) definition of the *R.decorata* group presented above. However, the snakes share the presence of a unique set of character states that distinguish them from all known species of *Rhadinaea* (see below). The new species may be known subsequently as:

### Rhadinaea nuchalis sp. n.

Taxon classificationAnimaliaSquamataColubridae

http://zoobank.org/0D170649-DD52-49A3-B331-34892F887ADF

**Figure 1.**
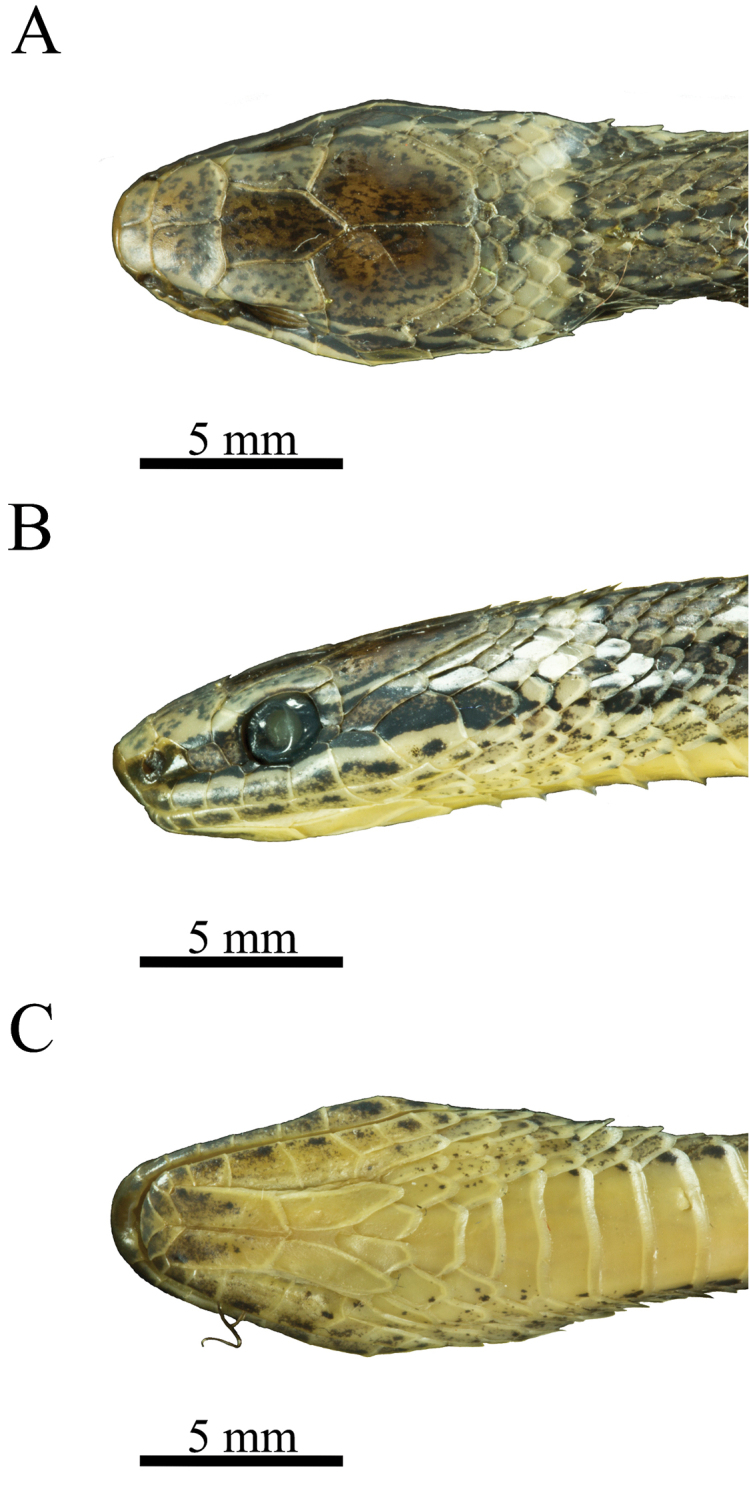
Head of *Rhadinaeanuchalis* sp. n. Holotype (MZFC-HE 22161) in dorsal (**A**), left lateral (**B**), and ventral (**C**) views.

**Figure 2.**
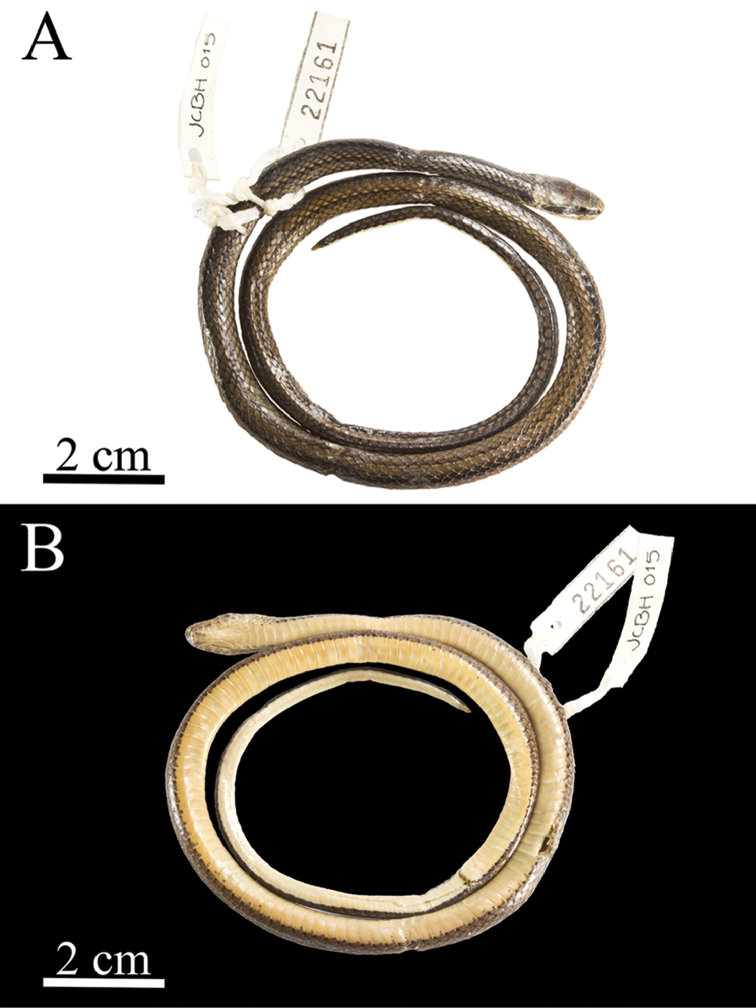
*Rhadinaeanuchalis* sp. n., holotype (MZFC-HE 22161). Dorsal (**A**) and ventral (**B**) views.

**Figure 3.**
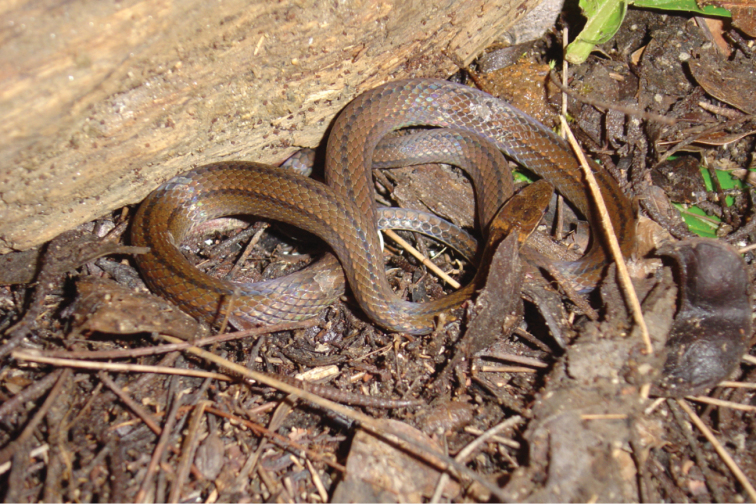
*Rhadinaeanuchalis* sp. n., holotype (MZFC-HE 22161) in life.

**Figure 4.**
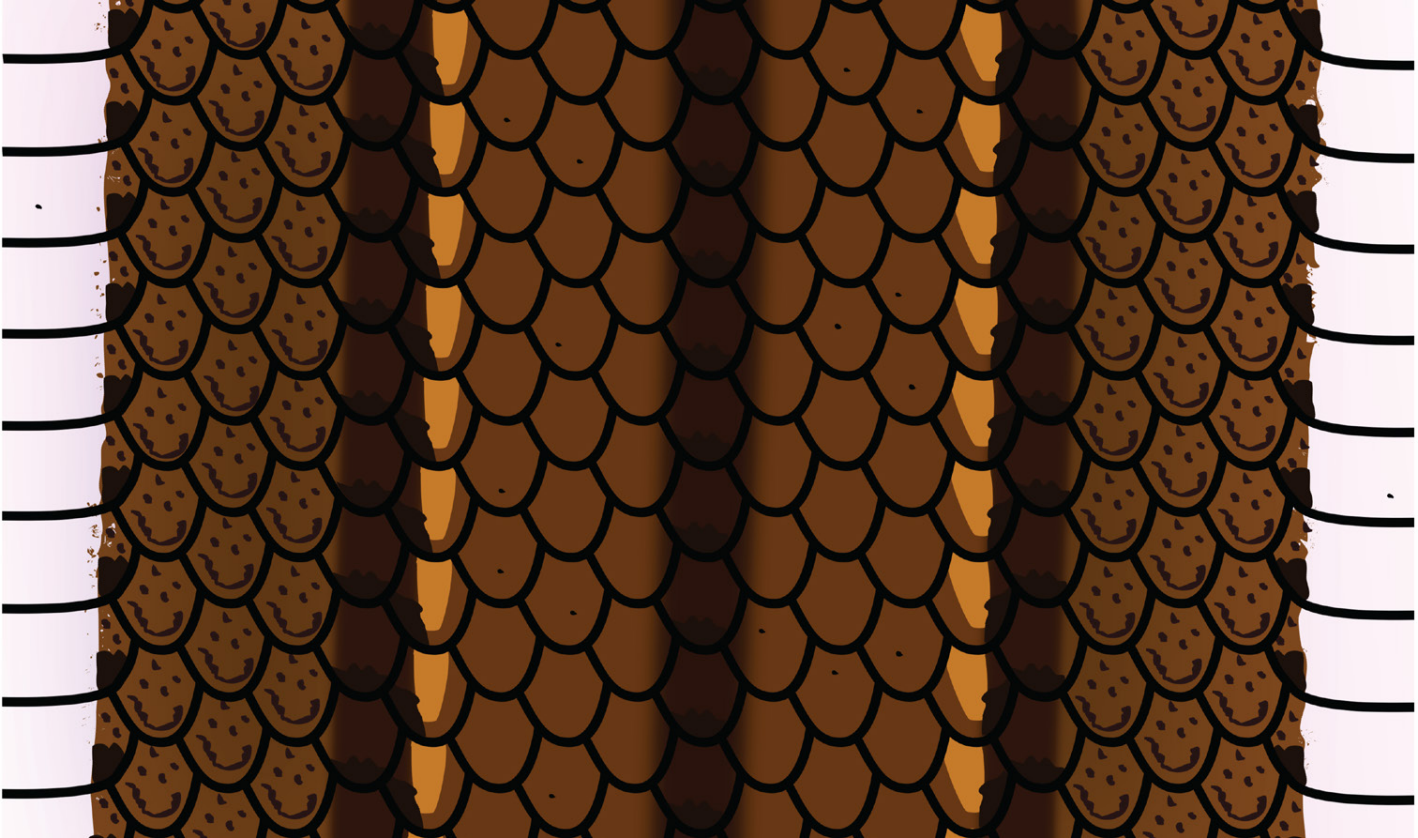
Diagram of coloration at level of midbody in *Rhadinaeanuchalis* sp. n. Based on holotype (MZFC-HE 22161).

**Figure 5.**
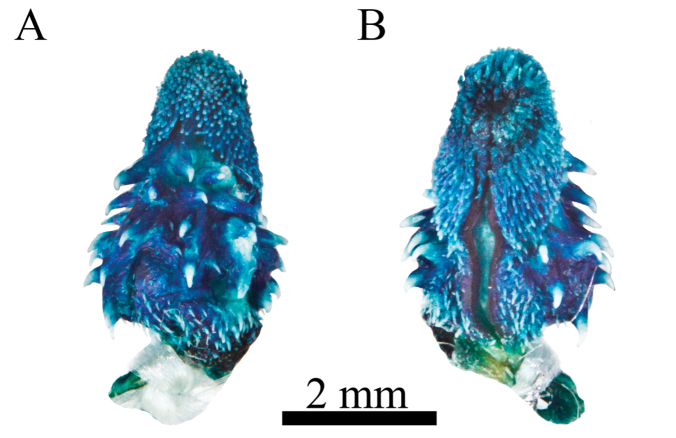
Right hemipenis of *Rhadinaeanuchalis* sp. n., holotype (MZFC-HE 22161). Asulcate (**A**) and sulcate (**B**) sides.

Figs 1 , 2 , 3 , 4 , 5

#### Type material.

**Holotype.** MZFC-HE 22161, (original field number JCBH 015) an adult male, from 0.36 km SE of El Molote, municipality of Atoyac de Álvarez, Guerrero, México (17.4167°N; 100.1672°W), ca. 1720 m elevation, collected by J.C. Blancas-Hernández on July 19, 2006. **Paratype.** MZFC-HE 34958, (original field number CIG 1078) an adult male, from El Molote, municipality of Atoyac de Álvarez, Guerrero, México (17.4376°N; 100.1891°W), ca. 1680 m elevation, collected by Christoph I. Grünwald, Héctor Franz-Chávez, and Karen I. Morales-Flores on September 11, 2016.

#### Diagnosis.

A colubrid snake of the *Rhadinaeadecorata* group (sensu Myers 1974) that may be distinguished from all other members of the genus *Rhadinaea* by the following combination of character states: eight supralabials; 149–151 ventrals in males; 63–77 subcaudals in males; presence of two large pale nuchal blotches, forming an incomplete collar that occupies two scales laterally and bissected along the dorsal midline; postocular pale marking consisting of a well-defined and narrow line beginning anteriorly behind the upper posterior margin of the eye and extending posteriorly nearly horizontally until connecting with the nuchal blotches; and ground color of the flanks extending to the lateral portion of the ventrals.

#### Comparison.

*Rhadinaeanuchalis* sp. n. may be distinguished from all other members of the genus *Rhadinaea* (except *R.cuneata* and some individuals of *R.hesperia* and *R.omiltemana* Günther, 1894) by the presence of two large pale nuchal blotches forming an incomplete collar that occupies two scales laterally and is bissected along the dorsal midline (pale nuchal marking one-scale long in *R.laureata* (Günther, 1868) and *R.marcellae*, absent in the other species). *Rhadinaeanuchalis* can be further distinguished from the members of the *R.flavilata* group by the presence of eight supralabials (usually seven in the *R.flavilata* group). Additionally, it differs from *R.calligaster* (Cope, 1875), *R.forbesi*, *R.hesperia*, *R.marcellae*, *R.macdougalli*, and *R.montana* by the presence of a well-defined, pale postocular line beginning anteriorly behind the upper posterior margin of the eye and extending nearly horizontally posteriorly until connecting with the nuchal blotches (pale postocular line oblique in *R.calligaster* [if present], *R.forbesi*, *R.macdougalli*, and *R.marcellae*; not connected with the pale post-cephalic markings in the other species [except for one side in one specimen of *R.montana*]).

Furthermore, *Rhadinaeanuchalis* can be distinguished from members of the *R.vermiculaticeps* group by having more ventrals in males (149–151 vs. 117–124 ventrals in males of the *R.vermiculaticeps* group). *Rhadinaeanuchalis* differs from *R.cuneata* by having less subcaudals in males (63–77 vs. 106–115) and by having a narrow postocular pale marking in the form of a nearly horizontal line (postocular pale marking wedge-shaped in *R.cuneata*). Specifically, *R.nuchalis* differs from other congeners that inhabit Guerrero except *R.myersi* by having fewer subcaudals in males (63–77 vs. 110–137 in *R.hesperia*; 85–90 in *R.omiltemana*; 91–121 in *R.taeniata* Peters, 1863). Additionally, it differs from *R.myersi*, *R.omiltemana*, and *R.taeniata* by having the dark ground color of the flanks extending to the lateral portion of the ventrals (dark ground color of the flanks not reaching ventrals in *R.omiltemana*, occasionally and faintly so in *R.myersi* and *R.taeniata*).

#### Description of holotype

(Figs 1, 2). Male; adult; head length = 12.2 mm, snout-vent length (SVL) = 275 mm, tail length = 104 mm. Head distinct from neck; snout long, contained 2.5 times in head length, rounded from above, projecting anteriorly beyond lower jaw; rostral broader than high, portion visible from above 0.6 times as long as its distance from frontal, 0.5 times as long as internasal common suture, upper edge slightly above level of upper margin of nostrils; internasals broader than long (width / length = 1.5), rounded laterally, contacting anterior and posterior nasals laterally, length and common suture ca. 0.8 and 0.4 times as long as prefrontal common suture, respectively; prefrontal contacting postnasal and loreal laterally, length ca. 0.3 times length of snout, common suture ca. 0.5 times frontal length; frontal longer than broad (width / length = 3.6/2.3), angulate posteriorly; supraocular large, contacting prefrontal, frontal, parietal, and upper postocular broadly, length ca. 1.3 times length of horizontal diameter of eye, 1.3/1.3 times as long as loreal, ventral margin projecting anteriorly and posteriorly beyond margins of orbit; parietals 1.6 times longer than broad, length approximately 0.4 times head length, common suture as long as frontal; nasal divided; prenasal 1.3/1 times as long as postnasal; prenasal and postnasal combined length ca. 2.3 times loreal length; loreal as high as long, contained 1.1 times in snout length, 0.4 times as long as horizontal diameter of eye, dorsal margin nearly straight; preocular single, 1.8 times higher than long; subpreocular present, tiny, separating preocular and fourth supralabial; postoculars two; upper postocular 1.4 times higher than long, 2.3 times longer than lower postocular; lower postocular 2.25 times longer than high; eye large, contained 4.1 times in snout length, vertical diameter 2.0 times distance from lip; supralabials 8/8, first and second contacting postnasal, second and third contacting loreal, fourth and fifth entering orbit, seventh largest, contacting anterior temporal, eight contacting lower posterior temporal; temporals 1 + 2; anterior temporal separating sixth and seventh supralabials from parietal; upper posterior temporals separated posteriorly by five nuchals; lower posterior temporal contacting anterior temporal and seventh supralabial anteriorly, eight supralabial ventrally. Mental 1.5 times broader than long, rounded anteriorly, separated from chinshields by first infralabials; infralabials 10/10, first to third contacting anterior chinshields, fourth to sixth separated from chinshields by interstitial skin, seventh to tenth separated by other scales; anterior chinshields 3.3 times longer than broad, as long as posterior chinshields; posterior chinshields separated from each other by two midgular scales.

Transverse dorsal scale rows 17-17-17, smooth; apical pits absent; ventrals 151; cloacal scute divided; paired subcaudals 63.

*Color* (in life; Figs 3–4). Dorsum of head ochre, extending to rostral anteriorly, to third dorsal scale posterior to parietals posteriorly along the dorsal midline, narrowing occupying one dorsal scale laterally; extending to upper half of nasal, lateral portion of prefrontal, upper third of preocular, lateral edge of supraocular, uppermost portion of upper postocular, lateral portion of parietal, and ventral portion of upper secondary temporal and dorsal scale posterior to it ventrolaterally; irregular dark markings present, except on ventral border anteriorl to orbit (creating the appearance of a faint preocular line). Postocular pale marking consisting of a nearly horizontal, black-bordered, cream line; beginning anteriorly on rear upper fourth of orbit; passing along upper portion of upper postocular, lateral portion of parietal, and upper portion of primary temporal, connecting with nuchal blotches posteriorly. Lateral stripe dark brown, black-bordered; ventrally bordering faint preocular line and postocular pale marking; extending anteriorly to posterior nasal, eighth supralabial posteriorly; occupying upper border of supralabials 1–6, upper half of supralabial 7, and entire surface of supralabial 8 except for anteroventral corner. Ground coloration below dark lateral stripe white, interspaces between each scale bright pink; supralabials 1–7, mental, infralabials, and anterior chinshields with irregular dark markings.

Nuchal blotches brownish orange; separated dorsally by median dark line; one dorsal scale long dorsally, widening laterally to two dorsal scales; nearly immaculate dorsally, with abundant dark speckling at level of supralabials; connected to pale ventral coloration. First and second transverse dorsal scale rows posterior to nuchal blotches reddish brown, slightly darker than rest of body.

Coloration of rest of body and tail: median dark line dark brown, with darker spots on tip of each dorsal scale in vertebral row; extending to third mid-dorsal scale posterior to parietals anteriorly, to tip of tail posteriorly; narrower anteriorly and posteriorly (confined to vertebral dorsal scale row near head and to medial edges of innermost dorsal scale rows in tail), wider at midbody (occupying vertebral dorsal scale row entirely and dorsal edges of adjacent dorsal scale rows). Dorsolateral stripe ochre; failing to contact nuchal blotches anteriorly by two scales, extending to tip of tail posteriorly; extending between upper portion of fifth and lower portion of eighth longitudinal dorsal scale rows at level of mid-body, between upper portion of second and lower portion of third longitudinal dorsal scale rows at level of mid-tail. Scales on fifth longitudinal dorsal scale row at level of mid-body and second at level of mid-tail exhibiting prominent orange spots. Lateral dark line bordering dorsolateral stripe ventrally; contacting nuchal blotches anteriorly, extending to tip of tail posteriorly; occupying three longitudinal dorsal scale rows just posteriorly to head, upper portion of fourth and lower edge of fifth longitudinal dorsal scale rows at level of mid-body, upper edge of first and lower edge of second longitudinal dorsal scale rows at level of tail. Flanks dark ochre, slightly darker than dorsolateral stripes, presenting abundant dark speckling. Ground color of flanks extending ventrally onto lateral portions of ventrals and subcaudals. Lateral portion of ventrals with black spots posteriorly at level of ventral edge of color of flanks. Conspicuous dark line passing along lateral edge of subcaudals. Remaining surface of ventrals and subcaudals white, suffused lightly with bright pink from head to level of mid-body, with sparse tiny dark dots.

*Hemipenes* (Figure 5). Hemipenes unilobed, unicapitate, length ≈ 5 mm. Sulcus spermaticus centripetal basally, centrolineal distally; bifurcating at level of distal end of basal third of capitulum, terminating distally at level of basal end of distal third of capitulum; sulcus spermaticus walls smooth, well-defined; intrasulcar region nude. Capitulum calyculate, longer on sulcate side (≈ 2.5 mm, vs. ≈ 1.2 mm on asulcate side); calyces papillate in most of capitulum, spinulate near base of capitulum. Hemipenial body covered in spines distally; enlarged spines 30, slightly curved, larger and more abundant on lateral surfaces of hemipenial body and asulcate side than on sulcate side; area of hemipenial body below spinose section covered in small spinules; spinules surrounding sulcus spermaticus walls, covering larger area on sulcate and asulcate sides than on lateral surfaces of hemipenial body, separated from enlarged spines by triangular nude patch on asulcate side. Basal-most portion of hemipenial body nude.

#### Variation.

The paratype differs from the holotype by having the upper posterior temporal divided into two small scales on the right side, 10/9 infralabials, the posterior chinshields separated from first ventral by two rows of small scales, 149 ventrals, and 77 subcaudals. No remarkable differences in color pattern are present in the paratype with respect to the holotype.

#### Etymology.

The specific name *nuchalis* comes from the Latin *nucha*, meaning nape. It makes reference to the large nuchal blotches present in the new species.

#### Distribution and ecology.

**Figure 6.**
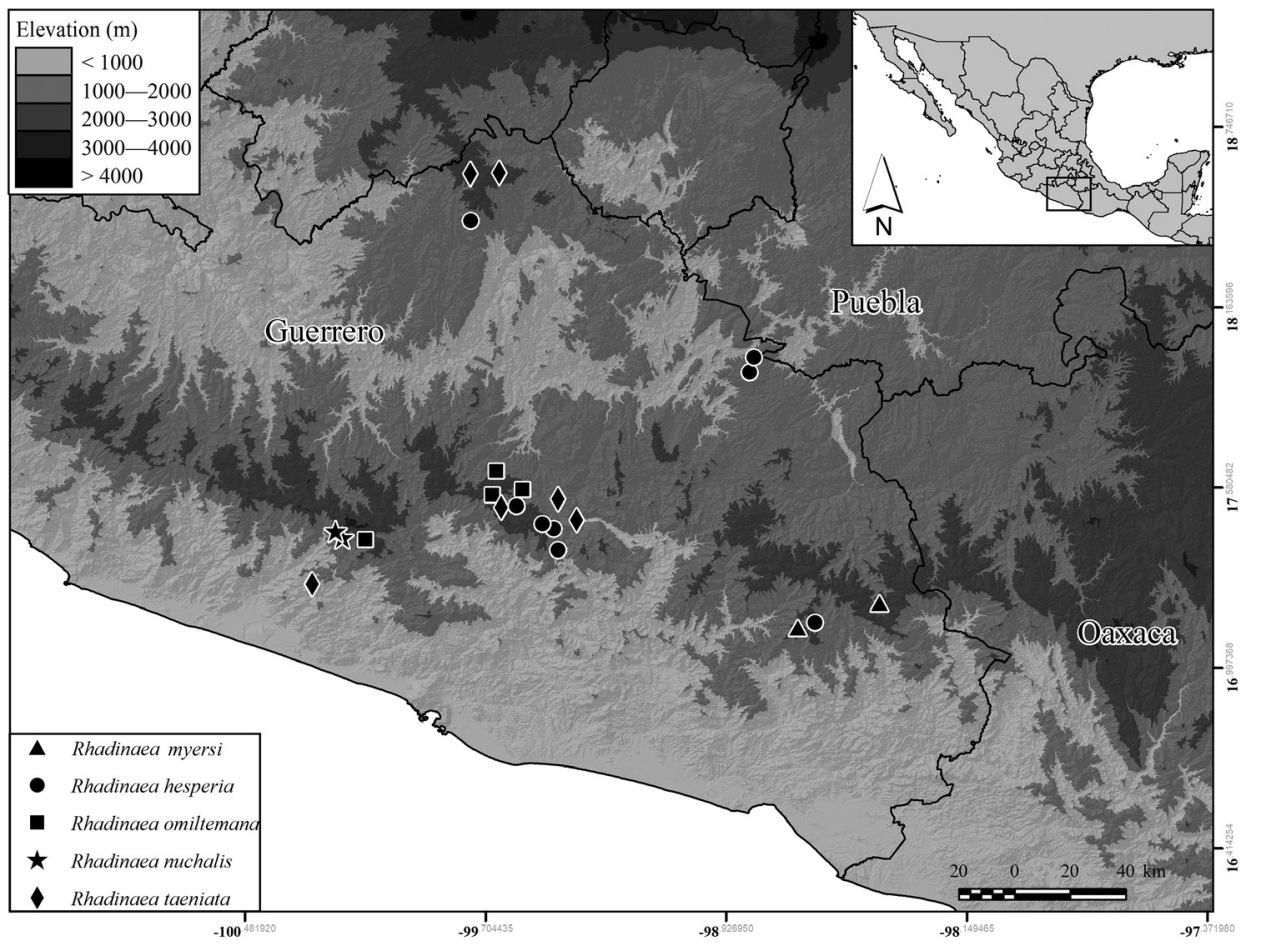
Collection localities of the species of the genus *Rhadinaea* in Guerrero. Black lines represent state limits.

*Rhadinaeanuchalis* sp. n. is known only at intermediate elevations from the vicinity of El Molote in the western portion of the Sierra Madre del Sur of Guerrero. The species appears to be allopatric with the other species of the *R.decorata* group. The closest *Rhadinaea* record to *R.nuchalis* is that of *R.omiltemana* from El Tambor, Coyuca de Benítez, Guerrero, approximately 8 km E in straight line from the type locality of *R.nuchalis* (Palacios-Aguilar et al. 2016). The second closest record is that of *R.taeniata* from 1.5 mi N San Vicente de Jesus, Guerrero, about 18 km SSW in straight line from the type locality of *R.nuchalis* (Myers 1974). Other close records are those of *R.omiltemana* from Omiltemi and Asoleadero, *R.hesperia* from Acahuizotla and Chilpancingo, and *R.taeniata* from Chilpancingo, all in Guerrero (Fig. 6). All the three are about 72 km, straight line, from the type locality of *R.nuchalis* The closest records of species in the *R.decorata* group, excluding those of *R.hesperia*, to the type locality of *R.nuchalis* are those of *R.myersi* from Malinaltepec, Guerrero, and southwestern Oaxaca (García-Vázquez et al. 2006); and those of *R.macdougalli* and *R.bogertorum* from northern Oaxaca (Myers 1974, Ramírez-Bautista et al. 1998).

**Figure 7.**
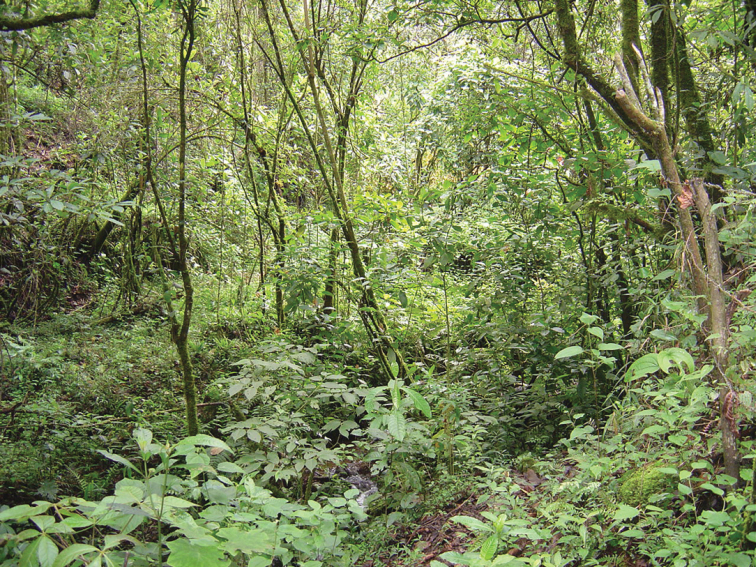
Habitat of *Rhadinaeanuchalis* sp. n. in the type locality.

The region of El Molote is characterized by rugged topography and the presence of numerous permanent streams that flow into the Atoyac and Coyuca rivers, whose basins belong to the Costa Grande hydrologic region (Lozada et al. 2003). Additional descriptions of the climate and other ecological aspects of El Molote can be found in Meza and López (1997) and Pavón-Vázquez et al. (2011). Coffee plantations have replaced most of the original cloud forest. The forest is dense and tall in undisturbed places, with the canopy reaching 25–30 m in height (Fig. 7). *Pinusayacahuite*, P.strobusvar.chiapensis, and *Ulmusmexicana* are emergent species, and *Alfaroacostaricensis*, *Sloanea* sp., *Quercussalicifolia*, *Cojobaarborea*, *Magnoliaschiedeana*, and *Zanthoxylummelanostictum* are dominant species (Lozada et al. 2003).

##### Identification key

An identification key to the species of *Rhadinaea* was included in Myers’s (1974) revision of the genus. Examination of the known specimens of *R.nuchalis* would lead to couplet number 40 in Myers’s (1974) key for North American species. Modifying number 40 as follows would allow the identification of *R.nuchalis*:

**Table:** 

40	Pair of large pale nuchal blotches, forming a collar broken on the dorsal midline	***R.nuchalis* sp. n.**
–	Post-cephalic pale markings not significantly enlarged, not as described above	**41**

Former number 40, a bracket including *R.bogertorum* and *R.myersi*, would become number 41.

## Discussion

Color pattern has been considered one of the most informative characters for distinguishing between species and species groups within *Rhadinaea*: “…once some idea has been gained of intraspecific variation, color pattern offers the most generally reliable method of identifying species because no two forms have identical patterns…” (Myers 1974). Our own examination of eleven species of *Rhadinaea*, including all of those distributed in Guerrero, and a review of the relevant literature for the whole genus revealed the existence of intraspecific variation in color pattern for some species, but not a single specimen of another species exhibits the combination of coloration characters present in *R.nuchalis*. Additionally, *R.nuchalis* can be distinguished from morphologically similar species, except for *R.forbesi*, and its geographically closest congeners by the presence of a low number of subcaudals in males (see Comparison), and from *R.forbesi* by having a greater number of ventrals in males (149–151, vs. 136–149 in males of *R.forbesi*).

DNA sequences of *Rhadinaea* have been included in studies looking at phylogenetic relationships above the generic level (e.g., Lawson et al. 2005, Pyron et al. 2013), but a molecular assessment of the monophyly of *Rhadinaea* (sensu Myers 2011) and its species groups is still pending. However, species groups within *Rhadinaea* are divergent morphologically and assignment of *R.nuchalis* to the *R.decorata* group is relatively straightforward based on external and hemipenial morphology (see above). Thus, the definition of the group provided by Myers (1974) does not need to be modified by the inclusion of *R.nuchalis*.

The presence of a continuous median dark line, the extension of the dark ground color of the flanks onto the ventrals, the darker coloration of the flanks with respect to the stripes surrounding the median dark line, the relative length of the capitulum and sulcus spermaticus, and the high number of hemipenial spines suggest that *Rhadinaeanuchalis* may be closely related to *R.hesperia* (Myers 1974), its geographically closest congener within the *R.decorata* group. However, *R.hesperia* can be differentiated from *R.nuchalis* by the number of subcaudals in males (see Comparison) and ventral coloration (white anteriorly, grading to deep reddish posteriorly vs. white, suffused lightly with bright pink from head to level of mid-body in *R.nuchalis*). Additionally, although *R.hesperia* occasionally has a pair of slightly enlarged white blotches on the neck, in all cases the pale postocular marking does not coalesce with them.

The congeners geographically closest to *Rhadinaeanuchalis* outside the *R.decorata* group are *R.omiltemana* and *R.taeniata*, members of the *R.taeniata* group. They differ from *R.nuchalis* by having more subcaudals in males; the dark ground color of the flanks usually not reaching the ventrals, except for some individuals of *R.taeniata* in which case the color is faint (see Comparison); a broad (involving at least five dorsal scale rows) dorsal stripe darker than the flanks (median dark line flanked by two stripes paler than the flanks and occupying only the vertebral row and the innermost portions of adjacent scale rows in *R.nuchalis*); and a relatively small capitulum, comprising between two-sevenths and two-fifths of the length of the sulcate side of the hemipenis (capitulum comprising approximately half of length of the sulcate side in *R.nuchalis*).

The Sierra Madre del Sur of Guerrero has received scant attention from herpetologists and most expeditions have focused on the central region of the state, particularly in the Chilpancingo region (i.e. Campbell and Armstrong 1979, Adler 1996). However, the west and east portions of the Sierra have been largely ignored. Recent visits to the west portion have yielded a number of discoveries of new species (e.g., Campbell and Flores-Villela 2008, Campbell et al. 2009, Pavón-Vázquez et al. 2011, Feria-Ortiz and García-Vázquez 2012, Campbell et al. 2014). This suggests that the diversity of the Sierra Madre del Sur of Guerrero is higher than currently known (Palacios-Aguilar and Flores-Villela 2018), particularly for small and secretive snakes as exemplified by the present work and the recent descriptions of *Epictiaschneideri* Wallach, 2016, *Geophisoccabus* Pavón-Vázquez, García-Vázquez, Blancas-Hernández & Nieto-Montes de Oca, 2011, and *Rhadinelladysmica* Campillo, Dávila-Galavíz, Flores-Villela & Campbell, 2016.

## Supplementary Material

XML Treatment for Rhadinaea nuchalis

## Supplementary materials

Supplementary material 1 Specimens examined Data type: specimens data This is an open access article distributed under the terms of the Creative Commons Attribution License (CC BY 4.0), which permits unrestricted use, distribution, and reproduction in any medium, provided the original author and source are credited.Uri Omar García-Vázquez, Carlos J. Pavón-Vázquez, Jean Cristian Blancas-Hernández, Epifanio Blancas-Calva, Eric Centenero-Alcalá
