# Supplementary material for: A new rare species of the Rhadinaeadecorata group from the Sierra Madre del Sur of Guerrero, Mexico (Squamata, Colubridae)
Source: Zookeys. 2018 Aug 8;(780):137–54. doi: 10.3897/zookeys.780.25593 (PMC6093967; doi:10.3897/zookeys.780.25593)
Supplement: Supplementary material 1 — Specimens examined [file zookeys-780-137-s001.docx]

**Supplementary material 1. Specimens examined.**

Acronyms for herpetological collections follow Sabaj (2016), except for MZFC-HE for the Museo de Zoología of the Facultad de Ciencias, Universidad Nacional Autónoma de México. AMH, ANMO, DGM, ECA, JCSG, OORH, and UOGV are field identifiers for uncatalogued specimens being deposited in the MZFC-HE. All specimens from Mexico.

| **Species** | **Voucher number** | **Locality** | **Latitude** | **Longitude** |
| --- | --- | --- | --- | --- |
| *Rhadinaea bogertorum* | ANMO 2299 | 2.5 km W Totontepec, Oaxaca | 17.5972°N | 96.4229°W |
|  | ANMO 2312–2313 | Santiago Comaltepec, Oaxaca | 17.5964°N | 96.4236°W |
|  | MZFC-HE 27182 | San Pedro Yolox, Oaxaca | 17.5972°N | 96.4229°W |
| *R. cuneata* | DGM 26 | Aticpac, Zongolica, Veracruz | – | – |
| *R. decorata* | ANMO 2265 | Metates, San Juan Bautista, Oaxaca | 17.6956°N | 96.3231°W |
|  | ECA 373–374 | Salvador Díaz Mirón, Misantla, Veracruz | – | – |
|  | JCSG 62 | La Campechana, Chontla, Veracruz | – | – |
| *R. forbesi* | MZFC-HE 5134 | 8 km (by air) SW Banderillas, Rancho El Álamo, Veracruz | – | – |
| *R. gaigeae* | AMH 674 | Ejido el Piñón, 15 km S Jacala, México Road 85 Jacala-Zimapán , Hidalgo | 20.9491°N | 99.2120°W |
|  | MZFC-HE 6228 | La Mojonera, Zacualtipán, Hidalgo | – | – |
|  | MZFC-HE 7785 | 3 km SE of El Madroño, Querétaro | – | – |
|  | MZFC-HE 8433 | 1 km NE El Doctor, Cadereyta de Montes, Querétaro | 20.8511°N | 99.5956°W |
|  | MZFC-HE 8434 | 1.2 km NE La Florida, Arroyo Seco, Querétaro | 21.4222°N | 99.7594°W |
|  | MZFC-HE 858 | Gloria- Las Palmas road, SW Estación Canindo, Tamaulipas | 20.1367°N | 98.1300°W |
|  | MZFC-HE 9165 | 7 km SE Tres Lagunas, Landa de Matamoros, Querétaro | 21.2967°N | 99.1794°W |
| *R. hesperia* | MZFC-HE 10427 | 8 km NW Caleta de Campos, Michoacán | – | – |
|  | MZFC-HE 11320 | 2 km S Mesa del Roble, Calvillo, Aguascalientes | 21.7514°N | 102.7622°W |
|  | MZFC-HE 11499 | Jicalán, Uruapan, Michoacán | – | – |
|  | MZFC-HE 12714 | Nuevo Urecho, Michoacán | 19.1639°N | 101.8700°W |
|  | MZFC-HE 2010 | La Primavera, Rio Caliente, Zapopan, Jalisco | – | – |
|  | MZFC-HE 3815 | Ixcateopan de Cuauhtémoc, Guerrero | – | – |
|  | MZFC-HE 6052 | El Grullo, Jalisco | – | – |
|  | MZFC-HE 801 | Cueva del Diablo, Ayala, Morelos | – | – |
|  | MZFC-HE 8285 | 2.3 km Road Terrero, Minatitlán, Colima | – | – |
|  | MZFC-HE 8286 | La Hacienda, Minatitlán, Colima | – | – |
|  | OORH 55 | 6 km N of Xochiatenco, Malinaltepec, Guerrero | 17.1446°N | 98.6397°W |
|  | UOGV 2100 | Barranca del Rio Santiago, Presa Colimilla, Jalisco | – | – |
| *R. marcellae* | ANMO 4339 | Finca Cruz Blanca, Xilitla, San Luis Potosí | 21.3946°N | 99.0000°W |
|  | MZFC-HE 5324 | Approximately 25 km (by air) NE Zacatlán, along Zaragoza-Huachinango road, Puebla | – | – |
|  | MZFC-HE 5868 | 5.3 km E Tlanchinol, Arroyo de Apantlazol, Hidalgo | 21.0000°N | 98.3500°W |
|  | UTA-R 12410–12416 | 4.5 km NE Tlanchinol, Hidalgo | – | – |
| *R. myersi* | MZFC-HE 19372 | Ejido Tres Marías, Malinaltepec, Guerrero | 17.1267°N | 98.6950°W |
|  | MZFC-HE 19373 | Road from Cerro de Vidrio to Santa Catarina Juquila, Santa Catarina Juquila, Oaxaca | 16.2289°N | 97.2564°W |
| *R. omiltemana* | MZFC-HE 2905–2911 | Chilpancingo de los Bravo, Omiltemi, Guerrero | – | – |
| *R. quinquelineata* | MZFC-HE uncatalogued | Hueyapan, Puebla | – | – |
| *R. taeniata* | MZFC-HE 12314 | Coalcomán, 1 km sureste Puerto La Zarzamora, Michoacán | – | – |
|  | MZFC-HE 14111, 23859 | Santa María Yavesia, Oaxaca | 17.2439°N | 96.4317°W |
|  | MZFC-HE 16631 | Avándaro, México | – | – |
|  | MZFC-HE 23862 | Santa María Yavesia, Oaxaca | 17.2200°N | 96.4356°W |
|  | MZFC-HE 23870 | Santa María Yavesia, Oaxaca | 17.2239°N | 96.4164°W |
|  | MZFC-HE 25624 | Tecolotlán, Jalisco | 20.2858°N | 104.0433°W |
|  | MZFC-HE 26875 | 13.2 km (by road) W of Mazatlán, Guerrero | 17.4358°N | 99.5394°W |
|  | MZFC-HE 27342–27343 | El Peñón, Valle de Bravo, México | – | – |
|  | MZFC-HE 2912–2915 | Chilpancingo de los Bravo, Omiltemi, Guerrero | – | – |
|  | MZFC-HE 3811–3812 | Cerro del Huizteco, Taxco de Alarcón, Guerrero | – | – |
|  | MZFC-HE 633 | Zumpango del Río, Guerrero | – | – |
|  | MZFC-HE 6803 | El Tepeixtle, 3 km N Terreros, Reserva de Manantlán, Colima | – | – |
|  | MZFC-HE 798 | Ayala, Morelos | – | – |
